# Efficacy evaluation of hydrogen peroxide disinfectant based zinc oxide nanoparticles against diarrhea causing *Escherichia coli* in ruminant animals and broiler chickens

**DOI:** 10.1038/s41598-024-59280-4

**Published:** 2024-04-22

**Authors:** Walaa I. Ahmed, Asmaa N. Mohammed, AL-Shimaa A. Sleim

**Affiliations:** 1 Bacteriology Lab., Alexandria Provincial Lab., Animal Health Research Institute, Agriculture Research Center (ARC), Giza, Egypt; 2 Department of Hygiene, Zoonoses and Epidemiology, Faculty of Veterinary Medicine, Beni-Suef University, Beni-Suef, 62511 Egypt

**Keywords:** ERIC–PCR fingerprint, Virulent *E. coli*, Disinfectants, Different animal species, ZnO NPs, Microbiology, Materials science

## Abstract

Different strains of *Escherichia coli* that exhibit genetic characteristics linked to diarrhea pose a major threat to both human and animal health. The purpose of this study was to determine the prevalence of pathogenic *Escherichia coli* (*E. coli*), the genetic linkages and routes of transmission between *E. coli* isolates from different animal species. The efficiency of disinfectants such as hydrogen peroxide (H_2_O_2_), Virkon®S, TH^4+^, nano zinc oxide (ZnO NPs), and H_2_O_2_-based zinc oxide nanoparticles (H_2_O_2_/ZnO NPs) against isolated strains of *E. coli* was evaluated. Using 100 fecal samples from different diarrheal species (cow n = 30, sheep n = 40, and broiler chicken n = 30) for *E. coli* isolation and identification using the entero-bacterial repetitive intergenic consensus (ERIC–PCR) fingerprinting technique. The *E. coli* properties isolated from several diarrheal species were examined for their pathogenicity in vitro. Scanning electron microscopy (SEM), high-resolution transmission electron microscopy (HR-TEM), Fourier-transform infrared spectrum (FT-IR), X-ray diffraction (XRD), zeta potential, and particle size distribution were used for the synthesis and characterization of ZnO NPs and H_2_O_2_/ZnO NPs. The broth macro-dilution method was used to assess the effectiveness of disinfectants and disinfectant-based nanoparticles against *E. coli* strains*.* Regarding the results, the hemolytic activity and Congo red binding assays of pathogenic *E. coli* isolates were 55.3 and 44.7%, respectively. Eleven virulent *E. coli* isolates were typed into five ERIC-types (A1, A2, B1, B2, and B3) using the ERIC-PCR method. These types clustered into two main clusters (A and B) with 75% similarity. In conclusion, there was 90% similarity between the sheep samples' ERIC types A1 and A2. On the other hand, 89% of the ERIC types B1, B2, and B3 of cows and poultry samples were comparable. The H_2_O_2_/ZnO NPs composite exhibits potential antibacterial action against *E. coli* isolates at 0.04 mg/ml after 120 min of exposure.

## Introduction

Diarrhea remains one of the leading causes of morbidity and mortality in developing nations^1^. The alarming problem with *E. coli* is the fact that pathogenicity is increased by the high prevalence rate and antibiotic resistance. Nonetheless, without adequate investigation, these issues cannot be addressed. Further research is required, particularly in developing nations such as South Africa^2^. *E. coli* belongs to the Enterobacteriaceae family and is a facultative anaerobic gram-negative rod-shaped bacterium. This bacteria, which is mostly fecal in origin, lives in the gastrointestinal tracts of both healthy and sick animals and humans. It is often discharged into the surrounding environment^3^.

Animals can serve as reservoirs for pathogenic *E. coli,* and these organisms can be transferred to humans via food consumption, water contamination with animal feces, and/or interaction with infected animals or their environment^4^. This is a significant worldwide health concern for both people and animals. Animal output is negatively impacted by a range of *E. coli* infections, particularly in poultry businesses. These conditions include hemorrhagic colitis, blood poisoning diarrhea, urinary tract infections, and abdominal sepsis^5,6^. This presents a major global health risk to both humans and animals. The appropriate application of sanitary protocols is one of the primary areas of concern in cattle production systems^7^. Between 70 and 95% of cases reported globally were found to have the pathogenic strain of *E. coli*. In addition, *E. coli* strains have a substantial financial impact and are a foremost cause of illnesses in the global chicken and poultry sectors^5^. Based on its antigenic composition, the species *E. coli* is separated serologically into serogroups and serotypes (somatic O antigens for serogroups and flagellar or H antigens for serotypes). The third class of antigens, known as capsular or K antigens, is expressed by a large number of strains and plays a crucial role in pathogenesis^8^.

The source and path of microbial contamination have been identified using a number of molecular typing techniques^9–11^. Selecting a suitable approach for bacterial genotyping is contingent upon various factors, including instrument availability, cost, speed, sensitivity, strengths, and user-friendliness of databases^12,13^. Because it is simpler, quicker, and less expensive than PFGE or MLST for determining the genetic similarity of bacterial strains, a straightforward PCR-based technique called ERIC has been extensively used^14^.

ERIC as a repeat sequence is seen in bacterial genomes^15^. Several bacterial isolates, including *E. coli*, can have their clonal variability evaluated using these molecular biological methods^16^. Intergenic repetitive units were identified first in *E. coli* and *Salmonella enterica serovar Typhimurium.* The study of infectious disease epidemiology now incorporates molecular biology methods^17^. highlight the importance of using PCR-based genotyping methods in conjunction with serotyping for epidemiological studies of highly pathogenic *E. coli* strains^18^.

Several effective disinfectants became crucial to use to prevent or impede the growth of microorganisms. Furthermore, novel methods for disinfection formulas with low residual levels, like hydrogen peroxide, are needed for the present-interest products^19^. Moreover, it has been discovered that combining H_2_O_2_ with other antibacterial agents increases their ability to penetrate bacterial cells and/or strengthens their oxidizing effect^20^. In addition, nano zinc oxide particles that pierce the cell wall are one of the antibacterial agents that limit the growth of bacterial infections due to oxidative stress damage^21^. Moreover, ZnO NP was discovered to have an antibacterial impact on Gram-negative bacteria, such as *K. pneumoniae* and* E. coli*^22^.

Therefore, the purpose of this work was to ascertain the prevalence rate of pathogenic *E. coli* in numerous diarrheic species (cows, sheep, and broiler poultry), and the virulence indicators (Congo red and hemolysis binding ability) of *E. coli*. As well, the genetic diversity of the most virulent strains of *E. coli* was characterized using ERIC-PCR. Furthermore, the degree of similarity among the isolates was determined by the development of a dendrogram, which allowed for the comparison of clusters produced by the examination of various sampling locations and evaluating the variety of potential sources of contamination. Finally, the efficiency of several disinfectants (H_2_O_2_, Virkon® S, and TH^4+^), nano zinc oxide, and H_2_O_2_/ZnO NPs composite against the most pathogenic *E. coli* strains was assessed. Consequently, the current study is beneficial in preventing the incidence of diarrheal causes and their impact on animal health, as well as the breakout of pathogenic *E. coli* in cattle, sheep, and broiler poultry farms.

## Materials and methods

### Materials

Buffered peptone water (Oxoid), MacConkey's agar (Oxoid; CM0115), Eosin Methylene Blue (Oxoid; CM 69), and Analytical Profile Index 20E (API 20E) systems were used for *E. coli* isolation and identification. *E. coli* antisera (polyvalent and monovalent O), and commercially available kits (Test Sera Enteroclon, Anti-Coli, SIFIN Berlin, Germany) for serological typing of *E. coli*. The QIAamp DNA Mini kit (Qiagen, Germany, GmbH) was used to extract DNA. Primers, Emerald Amp Max PCR Master Mix (Takara, Japan). agarose gel (Applichem, Germany, GmbH). A Genedirex 100–3000 bp DNA ladder H3 RTU (Genedirex, Taiwan) was used to determine the fragment sizes. The online program (https://planetcalc.com/1664/) was used to calculate the number of crossing elements and the similarity index (Jaccard/Tanimoto Coefficient) between all investigated samples. TH^4+^ (SoGeVal, France), Virkon®S (Antec International TD, UK), hydrogen peroxide (H_2_O_2_, 6^th^ October 3^rd^ Industrial Area, Egypt), and zinc oxide (Loba, Chemi, Pvt. Ltd, India) for ZnO NPs synthesis.

### Study site and animal population

This study was carried out in a private broiler poultry, cattle, and sheep farms located in Alexandria Governorates during the period from September 2022 until October 2023. In addition to broiler chickens (n = 30), it also included ruminant animals (n = 70) at various phases of production. The cleaning and disinfection programs implemented at the farms under investigation received no particular emphasis, and the overall hygienic conditions on these farms were moderately fair.

### Ethical statement

There are no experimental studies on either animals or human data in the manuscript. All methods used in this context were carried out in compliance with the rules and regulations that applied. The data gathered was all documented and statistically analyzed.

### Samples collecting

Using sterile cotton swabs, 100 fresh fecal samples were directly obtained under aseptic conditions from various diarrheal species [cows (n = 30), sheep (n = 40), and broiler chickens (n = 30)]. These samples were transferred on ice for 2 h until they reached the laboratory^23^. Following accurate identification, samples were sent immediately to the lab for additional microbiological analysis.

### Isolation and identification of pathogenic *E. coli*

Each sample gathered was pre-enriched in buffered peptone water (Oxoid) and incubated for twenty-four hours at 37 °C in an aerobic environment. Next, MacConkey's agar (Oxoid; CM0115) was inoculated with a loopful of each broth culture. Colonies that tested positive for lactose were subcultured into Eosin Methylene Blue (Oxoid; CM 69) and incubated at 37 °C for 24 h. Selected metallic green colonies were sub-cultured on nutrient agar slopes and thereafter moved to semisolid medium to be stored at 4 °C in preparation for identification. The following biochemical tests were employed: TSI, indol, citrate utilization, urease, and methyl red tests, and Analytical Profile Index 20E (API 20E) systems were used for *E. coli* confirmation. Gram staining technique was applied, and Gram negative short bacilli were selected^24^.

## Recognition of *E. coli* pathogenicity

### Haemolytic activity of virulent *E. coli*

Blood agar bases enriched with 5% sheep blood were streaked with *E. coli* isolates, and the mixture was incubated at 37°C for 24 h. Colonies that create clear hemolysis zones are considered positive^25^.

### Congo red binding activity of virulent *E. coli*

The isolates of *E. coli* were streaked over Congo red agar and cultured for 72 h at 37 °C. The response was noted at 18, 24, 48, and 72 h. The presence of red colonies after 72 h was noted as a favorable response and indicated biofilm-producing *E. coli*. Even after 72 h, negative colonies remained white or grey because they were unable to bind the dye^26^.

### Serological typing of *E. coli*

Using *E. coli* antisera (polyvalent and monovalent O), agar slants harboring the most pathogenic and generous growth of *E. coli* (n = 11) were submitted for agglutination testing. Morris et al.^27^state that serological identification was used to identify *E. coli.* All isolates were serotyped in the Animal Health Institute's Serology Department using commercially available kits (Test Sera Enteroclon, Anti-Coli, SIFIN Berlin, Germany).

### ERIC-PCR characterization of pathogenic *E. coli*

The QIAamp DNA Mini kit (Qiagen, Germany, GmbH) was used to extract DNA from bacterial cells of fecal samples, with certain changes made in accordance with the manufacturer's instructions. In summary, 200 µl of the bacterial suspension was treated for 10 min at 56 ° with 10 µl of proteinase K and 200 µl of lysis buffer for the degradation and digestion of proteins. 200 µl of 100% ethanol was added to the lysate following incubation. After that, the sample was centrifuged and cleaned in accordance with the manufacturer's instructions. An elution buffer containing 100 µl was used to elute the nucleic acid. The oligonucleotide primers that were recorded in Table 1. For PCR amplification, primers were used in a 25 µl reaction that included 12.5 µl of Emerald Amp Max PCR Master Mix (Takara, Japan), 1 µl of each primer at a concentration of 20 pmol, 5.5 µl of water, and 5 µl of DNA template for PCR amplification. A 2720 thermal cycler from Applied Bio-system was used to carry out the reaction. The PCR products were separated by electrophoresis employing gradients of 5V/cm on a 1.5% agarose gel (Applichem, Germany, GmbH) with ethidium bromide staining in 1 × TBE buffer at room temperature. Twenty microliters of the items were put into each gel slot for the gel analysis. A Genedirex 100–3000 bp DNA ladder H3 RTU (Genedirex, Taiwan) was used to determine the fragment sizes. UV, or visible light is used by a gel documentation system (Alpha Innotech, Biometra) to stimulate fluorescent or chromogenic molecules in the gel. After the molecules produce light, an image is captured and saved by a camera. Computer software was then used to analyze the data^28^. Depending on whether each band was present or absent, the ERIC fingerprinting data was converted into a binary code. Ward's hierarchical clustering procedure and the unweighted pair group technique with arithmetic average (UPGMA) and SPSS, version 22, were used to cluster analysis and create dendrograms^29^. The online program (https://planetcalc.com/1664/) was used to calculate the number of crossing elements and the similarity index (Jaccard/Tanimoto Coefficient) between all investigated samples.

**Table 1:** Primer sequence, target gene, amplicon sizes and cycling conditions.

Target	Primers sequence	Amplified segment (bp)	Primary denaturation	Amplification (35 cycles)			Final extension	Reference
				Secondary denaturation	Annealing	Extension		
ERIC	ATG TAA GCT CCT GGG GAT TCA C	Variable	94 °C	94 °C	52 °C	72 °C	72 °C	Versalovic et al.^28^
	AAG TAA GTG ACT GGG GTG AGC G		5 min	30 s	1 min	1 min	10 min	

### Synthesis and characterization of tested ZnO NPs and H_2_O_2_/ZnO NPs

The method of high-energy ball milling (HEBM) was used to generate ZnO NPs^31^. Subsequently, to create H_2_O_2_ capping on ZnO NPs, 3% hydrogen peroxide was added to the various ZnO NP concentrations (0.02 and 0.04 mg/mL) right before use. The mixture was then vigorously shaken on a magnetic stirrer to minimize NP agglomerations throughout the incubation times (30, 60, and 120 min). SEM (JEOL (JSM-5200), Japan), HR-TEM (a JEOL JEM 2000EX), FT-IR (VERTEX, 70), XRD (PANalytical Empyean, Sweden), zeta potential, and distribution of particle size (A Malvern Instruments Ltd., Worcestershire, UK) were used to characterize both nano zinc oxide and H_2_O_2_/ZnO NPs. In the Central Lab of the Agriculture Faculty at Cairo University, Egypt, HR-TEM, and SEM micrographs were done. While at Beni-Suef University's Faculty of Postgraduate Studies of Advanced Science, the nanocomposite's FTIR spectra, XRD, particle size distribution, and zeta potential were achieved.

### Assessing antimicrobial method of disinfectants, ZnO NPs, and H_2_O_2_/ZnO NPs composite

Broth macro-dilution method was utilized to estimate the antibacterial efficacy of tested compounds. 100 µl of various bacterial strains (1 × 10^−6^ CFU/ml) were inoculated with 0.5% and 1% of TH^4+^ disinfectant (SoGeVal, France),Virkon®S (Antec International TD, UK) at the same concentrations, hydrogen peroxide (H_2_O_2_, 6th October 3rd Industrial Area, Egypt) at a concentration of 3 and 5%, ZnO NPs (0.02 and 0.04 mg/ml), and H_2_O_2_/ZnO NPs composite (0.02 and 0.04 mg/ml) in Mueller–Hinton broth (MHB) onto a 96-well plate (Sarstedt, Numbrecht, Germany) was evaluated against thirty strains of *E. coli* isolates according to Li et al.^30^ at different concentrations and testing times (30 min, 60 min, and 120 min). In order to generate the negative control, one microliter of broth culture was introduced to MHB without any testing materials. As a positive control, tested disinfectants and nanomaterials in MHB was conducted concurrently. A standard strain of *E. coli* ATCC 25,922 was applied as a quality control-positive organism. For 24 h, all of the tested materials were incubated at 37 °C. Three duplicates were used for the in-vitro experiment. In accordance with **CLSI**^32^ recommendations, one loopful of each well was inoculated on Mueller–Hinton agar to monitor the presence or lack of microbial growth at various doses of the tested substances.

### Statistical analysis

After being gathered, all of the data was entered into a Microsoft Excel spreadsheet to become available for analysis. Non-parametric tests (Chi-square test, K independent sample) using SPSS (statistical package for social sciences, version 22.0) were applied to determine the prevalence rate of pathogenic *E. coli* isolated from various diarrheal species, sero-grouping of some isolated strains, cluster analysis and dendrogram construction, and the bactericidal effect of testing disinfectants and nanocomposite against pathogenic *E.* coli, with a probability level of *p* ≤ 0.05.

## Results

The prevalence rate of pathogenic *E. coli* isolated from various diarrheal species was 38/100; 38% at (χ^2^) = 94, *P* ≤ 0.05. Additionally, the highest incidence rate of *E. coli* was found in diarrheal broiler chickens (13/30; 43.3%), followed by diarrheal sheep (15/40; 37.5%), and cows (10/30; 33.3%), as shown in Table 2.

**Table 2:** Prevalence rate of pathogenic *E. coli* isolated from different diarrheic species.

Samples collecting (fecal samples)	Total examined No	Prevalence rate of pathogenic *E.coli* isolates (No. %)	
		No	%
Cows	30	10	33.3
Sheep	40	15	37.5
Broiler chickens	30	13	43.3
Total	100	38	38

The chi-square association of prevalence rate of pathogenic *E. coli* isolates in collected samples is statistically significant at (χ^2^) = 94, *P* ≤ 0.05.

The hemolytic activity of all identified strains from diarrheal spp. was 21/38; 55.3%, according to the beta-hemolytic activity of pathogenic *E. coli* recovered from various diarrheal species. *E. coli* strains isolated from diarrheal cows (6/10; 60%) showed the highest hemolytic activity, followed by diarrheal broiler chickens and sheep (7/13; 53.8%) and 8/15; 53.3%, respectively, that were significantly different at (χ^2^) = 114, *P* ≤ 0.05. On the other hand, as Table 3 illustrates, *E. coli* isolates from diarrheal sheep (7/15; 46.6%) and broiler chickens (6/13; 46.1%) demonstrated CR positivity with varying degrees of red color.

**Table 3:** Pathogenicity determinants of *E. coli* isolates from different diarrheic species.

Diarrheic spp. (No.)	Pathogenicity determinants of *E*. *coli* (No. %)					
	Hemolytic activity		Congo red binding assay		Hemolytic activity& Congo red binding assay	
	No*	%	No*	%	No.*	%
Cows (*n* = 10)	6	60	4	40	4	40
Sheep (*n* = 15)	8	53.3	7	46.6	5	33.3
Broiler chickens (*n* = 13)	7	53.8	6	46.1	2	15.4
Total (n** = 38)	21	55.3	17	44.7	11	28.9

Pathogenicity determinants of *E. coli* isolates among diarrheic species was significantly different at (χ^2^) = 114, P ≤ 0.05.

*% according to total number of *E. coli* isolates recovered from each species.

** % according to total number of *E. coli* isolates recovered from all species.

**Figure 1:**
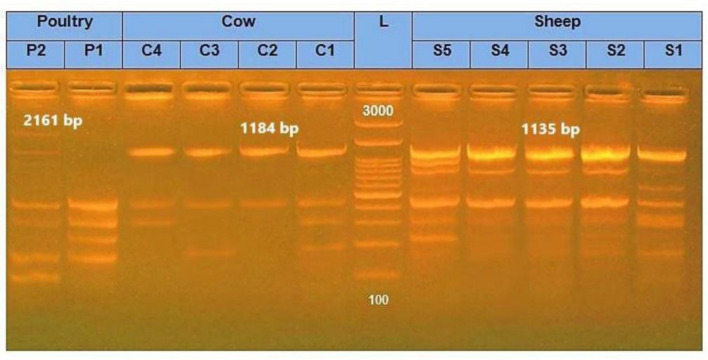
ERIC-PCR of the most virulent* E. coli* strains on agarose electrophoresis gel (1.5%) with ethidium bromide staining. Lane L: 100 bp Ladder (DNA MW marker). Lane S1, S2, S3, S4, and S5 (Sheep isolates) at a band of 1135 bp; Lane C1, C2, C3, and C4 (Cows isolates) at a band of 1184 bp; and Lane P1 and P2 (Poultry isolates) at a band of 2161 bp.

Utilizing DNA fragments obtained through isolated *E. coli* bacteria from sheep, cows, and broiler chickens, the variety and quantity of bands generated from electrophoresis on gels were noted. A range of 0 to 60 bands covering 70 bp to 2161 bp was found in the ERIC-PCR band sequences. It was found that isolated strains from sheep had the greatest frequency and variety. Moreover, strains isolated from chickens showed the highest degree of similarity among DNA molecule band patterns. The isolated strains from sheep, cows, and broiler chickens’ fecal samples showed prominent fragment sizes in DNA fingerprints of 1135 bp, 1184 bp, and 2161 bp, respectively; the observed bands, as illustrated in Fig. 1, ranged widely from 70 to 2161 bp. The serotyping of certain *E. coli* isolates obtained from various diarrheal species, as displayed in Table 4, showed that 11 (100%) of the isolated *E. coli* strains were typable. The most prevalent *E. coli* serogroup was O26:K60 (3), which was followed by O44:K74(2), O124:K72(2), O25:K11(2), O118: K-(1), and O78: K-(1).

**Table 4:** Serotyping of some *E. coli* isolates recovered from diarrheic species.

*E. coli* serogroups (*n* = 11)				Cows (*n* = 4)		Sheep (*n* = 5)		Broiler chickens (*n* = 2)	
Serogroup	Strain character	No	%*	No	%**	No	%**	No	%**
O26:K60	EHEC	3	27.27	ND	ND	3	60.0	ND	ND
O44:K74	EAggEC	2	18.2	ND	ND	2	40.0	ND	ND
O124:K72	EPEC	2	18.2	2	50.0	ND	ND	ND	ND
O25:K11	EPEC	2	18.2	2	50.0	ND	ND	ND	ND
O118:K-	EHEC	1	9.1	ND	ND	ND	ND	1	50.0
O78:K-	APEC	1	9.1	ND	ND	ND	ND	1	50.0

EHEC: Entero-hemorrhagic *E. coli*; EAggEC: Entero-aggregative *E. coli*; EPEC: Entero-pathogenic* E. coli*; APEC: Avian pathogenic *E. coli.*

ND: Not detected.

* Percentage according to total number of *E. coli* isolates.

**Percentage according to total number of *E. coli* isolates per species.

**Figure 2:**
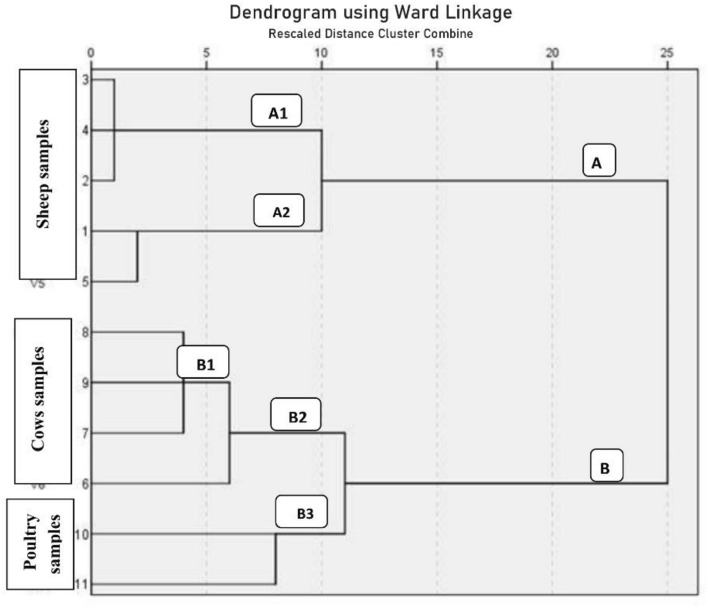
ERIC-PCR, dendrogram analysis shows genetic relationships among fecal *E. coli* isolates from sheep (A1 and A2), cows (B1 and B2), and poultry (B3).

**Figure 3:**
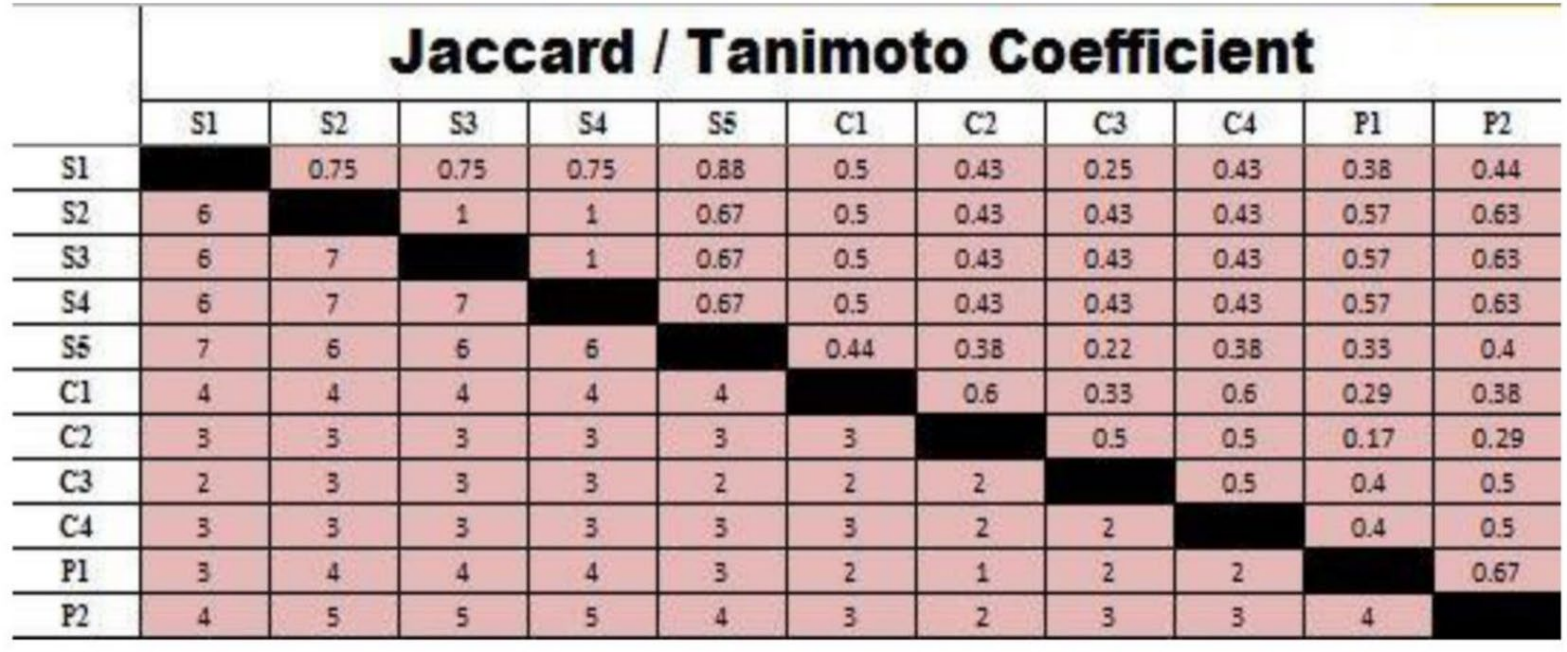
Genetic Similarity index of eleven virulent *E. coli* isolates.

In the present investigation, eleven virulent *E. coli* isolates were typed into ERIC-types using ERIC-PCR profiles. Using a 75% similarity limit, dendogram analysis separated them into two large clusters, A and B. Cluster A is separated into two groups, A1 and A2, containing five isolates that are sheep-related. The distribution of *E. coli* isolate numbers in group A1 is "3, 4, and 2", while in group A2 it is "1 and 5", respectively. Ninety percent of these two groups were comparable. With 6 isolates (cows (n = 4), which included *E. coli* isolates number "8, 9, 7, 10'') and broiler chickens (n = 2), which contained *E. coli* isolates number "10 and 11"), Cluster B was classified into groups (B1, B2, and B3). There was an 89% similarity between these three groups. Furthermore, for B1, B2, and B3, the similarity within each group was 96%, 94%, and 92%, respectively (Fig. 2). All *E. coli* isolates had an identity range of 0.17 to 1, but samples from sheep, cows, and broiler chickens had ranges of 0.67–1, 0.22–0.6, and 0.67–0.17, respectively (Fig. 3).

The antimicrobial sensitivity profile of testing disinfectants (TH^4+^, Virkon®S, and H_2_O_2_), ZnO NPs, and H_2_O_2_/ZnO NPs composite against pathogenic *E. coli* in Table 5 clarified that all isolated pathogenic* E. coli* and the control-positive strain (*E. coli* ATCC 25,922) were found to be completely sensitive to TH^4+^ at a concentration of 1:100 ml after 120 min of exposure time at *P* ≤ 0.05. In addition, the sensitivity of *E. coli* did not exceed 70% at the least concentration (1:200 ml) after 120 min of contact time. Conversely, Virkon®S disinfectant proved to be 100% effective against *E. coli* and *E. coli* ATCC 25,922 at a dosage of 1:100 ml after 120 min of contact time at *P* < *0.05.* In contrast, the sensitivity testing of *E. coli* isolates to H_2_O_2_ was significantly low at different contact times and did not exceed 50% at 5% concentration after time exposure (120 min) at *P* ≤ 0.01 compared to the lowest concentration of 3%. Oppositely, nano zinc oxide was verified to have a lethal effect (100%) on *E. coli* and a control positive stain at 0.04 mg/ml after 120 min. It's interesting to note that employing nano zinc oxide increases hydrogen peroxide's ability to penetrate bacterial cells. In comparison to other doses, it was discovered that hydrogen peroxide loaded on ZnO NPs was highly effective (100%) against all *E. coli* isolates and the control positive one at 0.04 mg/ml after 120 min of exposure compared to other concentrations.

**Table 5:** Antimicrobial efficiency of testing disinfectants and nanomaterials against pathogenic *E. coli.*

Testing compounds (Conc.)	Sensitivity profile of pathogenic *E. coli* (*n* = 30) isolates at varies exposure times			
	30 min	60 min	120 min	P value
TH^4+^				0.05
1:100 ml	60	60	100	
1:200 ml	40	50	70	
Virkon®S				0.03
1:100 ml	50	70	100	
1:200 ml	30	50	70	
H_2_O_2_				0.01
5%	20	30	50	
3%	0.0	10	30	
ZnO NPs				0.05
0.04 mg/ml	60	80	100	
0.02 mg/ml	60	70	80	
H_2_O_2_/ZnO NPs				0.02
0.04 mg/ml	90	90	100	
0.02 mg/ml	70	80	90	

**Figure 4:**
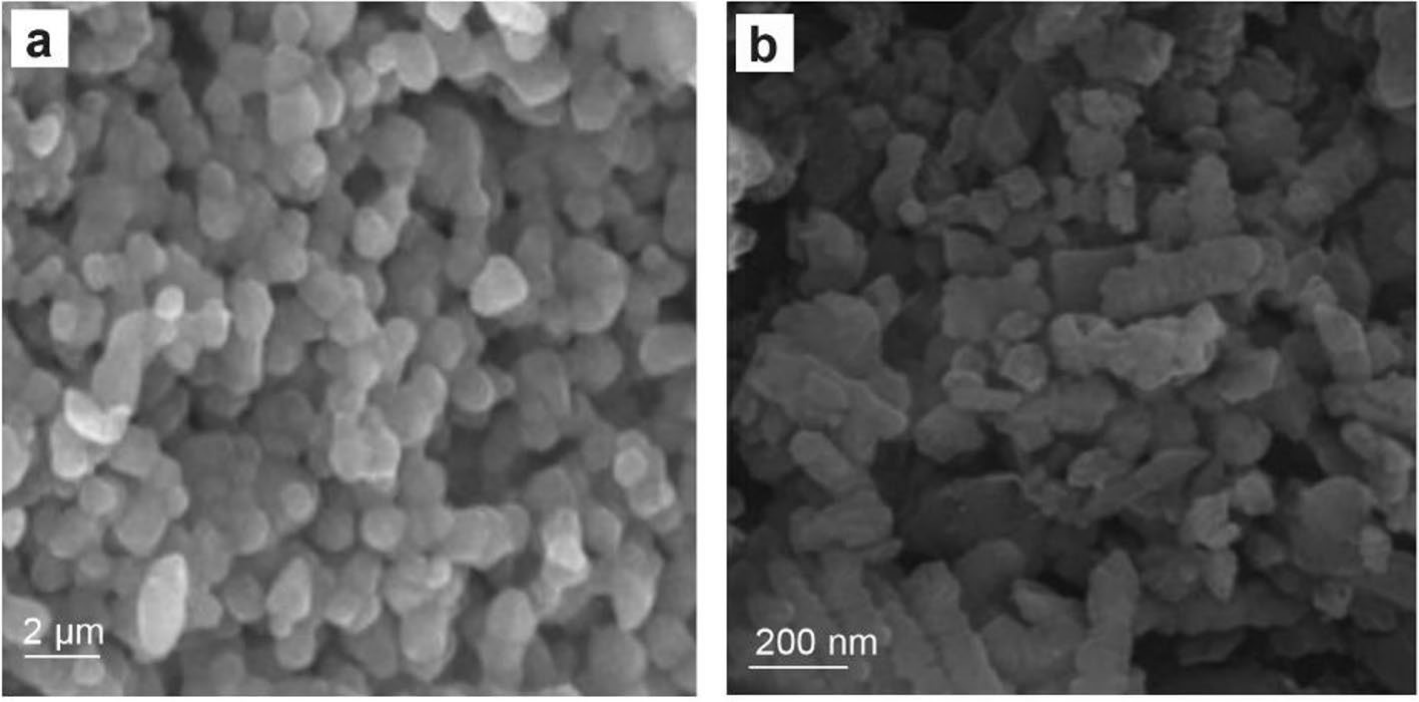
SEM microscopy of ZnO NPs (**a**) and H_2_O_2_/ZnO NPs composite (**b**).

**Figure 5:**
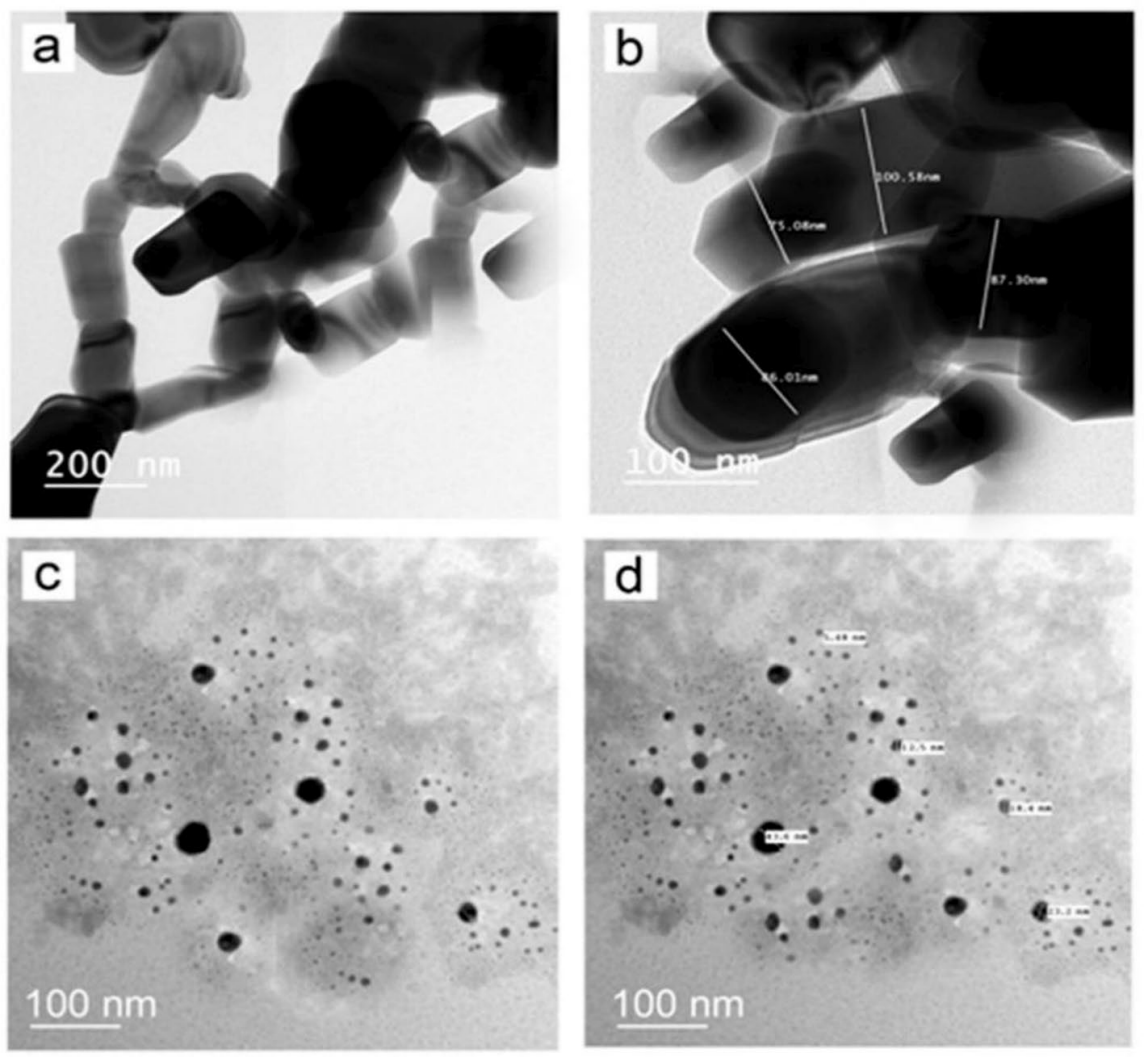
Transmission electron microscopy of ZnO NPs (**a-b**) clarified the hexagonal shape of zin oxide nanoparticles (**a**) and the diameter of NPs was ranged between 75.08 to 100.58 nm (**b**). Moreover, H_2_O_2_/ZnO NPs Micrographs exhibited the alteration in NPs shape to pentagonal (**c**) and the size of NPs in diameter was ranged from 5.48 to 34.6 nm (**d**).

**Figure 6:**
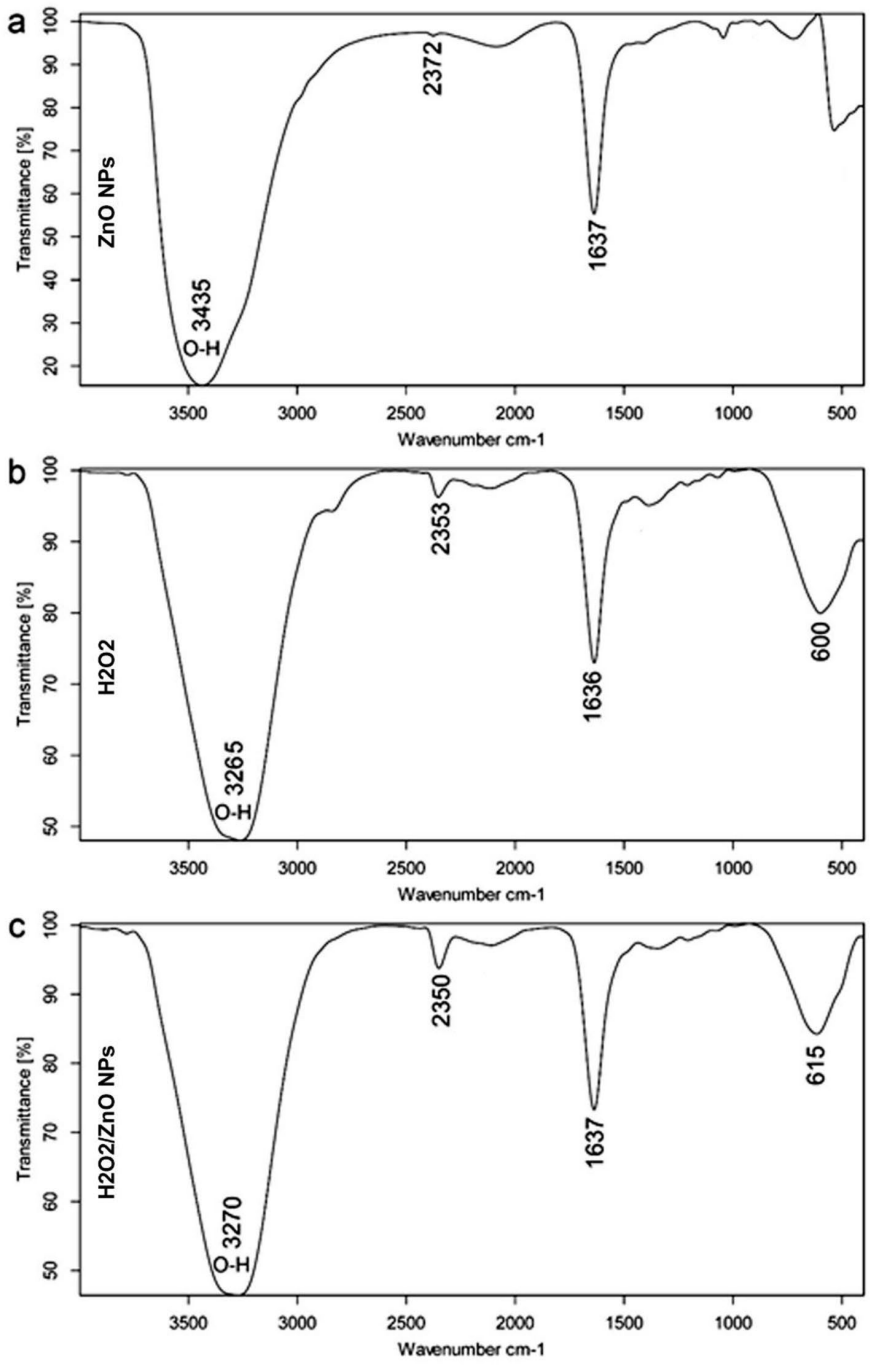
FTIR spectra of ZnO NPs (**a**), H_2_O_2_ (**b**), and H_2_O_2_/ZnO NPs composite (**c**).

**Figure 7:**
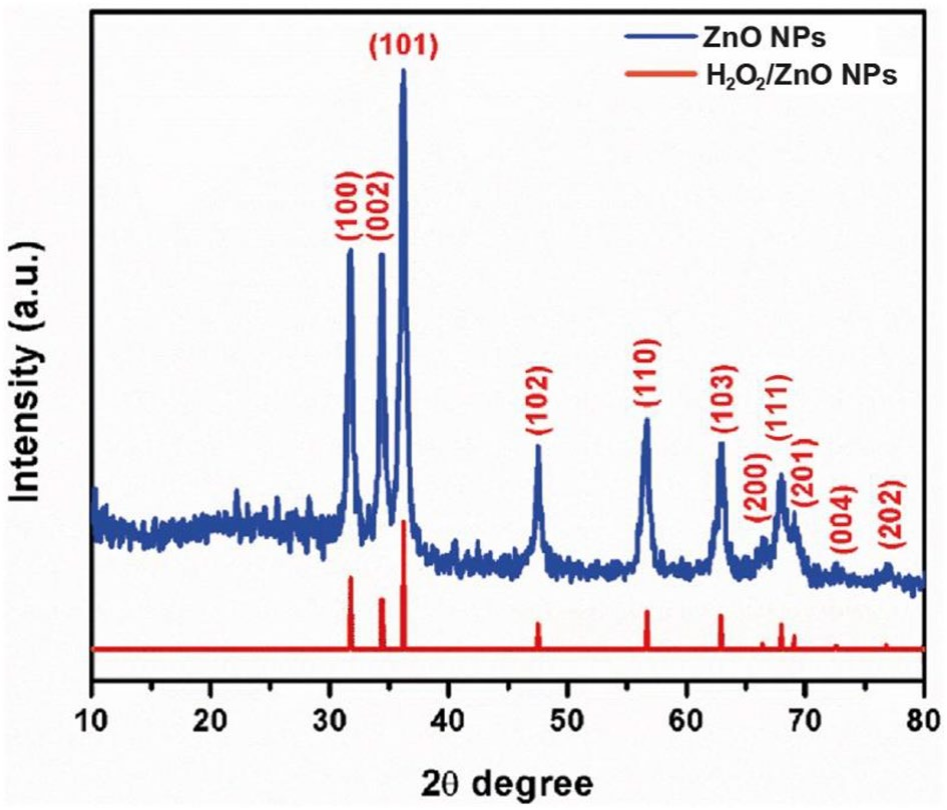
XRD pattern of ZnO NPs, and H_2_O_2_/ZnO NPs composite.

**Figure 8:**
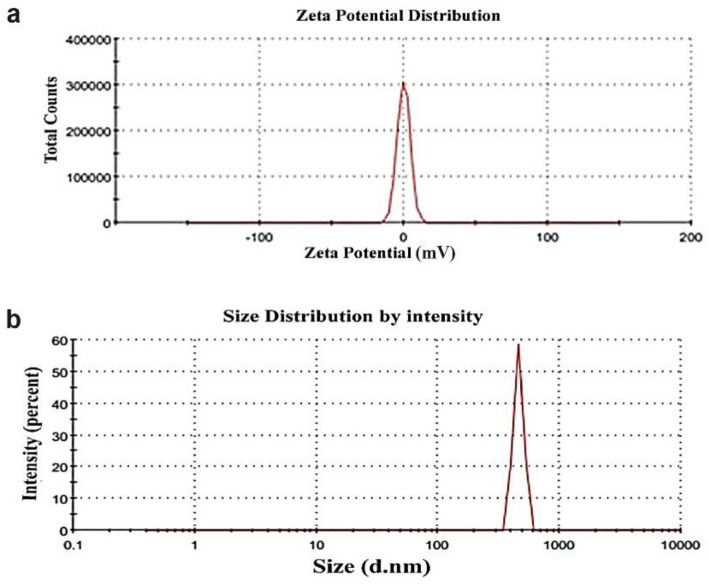
Zeta potential (mV) and particle size distribution (d. nm) of H_2_O_2_/ZnO NPs composite.

SEM microscopy of ZnO NPs, as shown in Fig. 4a. It emerged as uniform, spherical particles loaded on top of one another. After loading, H_2_O_2_/ZnO NPs (Fig. 4b) seemed to be a lot of elongated particles in shape. The morphological feature of nano zinc oxide (Fig. 5a) was revealed to be hexagonal, and the diameter of the NPs ranged from 75.08 to 100.58 nm (Fig. 5b), according to TEM microscopy. Additionally, TEM micrographs of H_2_O_2_/ZnO NPs revealed that the nanoparticles' shape had changed to a pentagonal form (Fig. 5c), and their diameter ranged from 5.48 to 34.6 nm (Fig. 5d). On the other hand, FTIR spectra of the hydrogen peroxide, nano zinc oxide, and H_2_O_2_ loaded on ZnO NPs, as shown in (Fig. 6) clarified that nano zinc oxide exhibited strong absorption peaks at 3435, 2372, 1637, 1044, 723, and 535 cm^−1^ (Fig. 6a). H_2_O_2_ revealed a wide range of absorption peaks linked to the absorption of hydroxyl groups (O–H). Moreover, characteristic peaks were observed at 3265, 2353, 2122, 1636, 1387, 1210, and 600 cm^−1^, respectively (Fig. 6b). Additionally, the composite H_2_O_2_/ZnO NPs (Fig. 6c) demonstrated the strongest peak migrated to 3270 and 2350 cm^−1^, in addition to characteristic stretching mode vibration peaks at 1346 and 615 cm^−1^, confirming the interaction between the tested disinfectant (H_2_O_2_) and nano zinc oxide. The structural properties of ZnO NPs, and H_2_O_2_/ZnO NPs composite were examined through XRD diffraction, as displayed in Fig. 7. The XRD pattern of ZnO NPs exhibited high crystallinity, where the presence of 100, 002, 101, and 110 planes matched the hexagonal crystal structure of nano zinc oxide. Besides, the intensity of peaks decreased in H_2_O_2_/ZnO NPs, exhibiting a decrease in the crystallinity of the composite. Oppositely, the stability and nanoparticle charge were measured using zeta potential (Fig. 8) based on their electrophoretic mobility. H_2_O_2_/ZnO NPs composite (Fig. 8a) had a negative charge of − 0.12 mV, and the hydrodynamic diameter of the particle size was 2625 nm (Fig. 8b).

## Discussion

Globally, enterotoxigenic *E. coli* (ETEC) bacteria are acknowledged as a significant contributor to the general issue of diarrhea^33^. Cattle are a natural reservoir for *E. coli* in livestock; where the bacteria are always carried in their feces and can infect anywhere from 1 to 50% of healthy cows^34^*.* Preventing an *E. coli* outbreak can be achieved by regularly monitoring of animals and enforcing strict hygiene measures during every stage of production and carried out at every stage of the supply chain, from farms to the employees who handle the animals. Rural farmers should look into the details and become more knowledgeable about different diets, their components, and the application of antibacterial agents. In emerging nations, epidemiological and pathogenic characteristics linked to the *E. coli* strain require more examination. Regular examinations of this pathogen are also necessary, particularly in urban and rural areas^35,36^.

*Escherichia coli* is one of the model organisms that is most thoroughly investigated^37,38^. ERIC-PCR is one of many techniques used to determine bacterial transmission. Various studies have employed it for a variety of bacterial isolates, including *E. coli, Salmonella spp., Pseudomonas aeruginosa,* and *Streptococcus*^39–41^*.* The current investigation found that 38 isolates out of 100 samples from various diarrheic species (cows, sheep, and broiler chickens) contained *E. coli*, with a total prevalence of 38%. In contrast, the prevalence rates in each of the diarrheal species were 33.3%, 37.5%, and 43.3%, respectively, as shown in Table 2. This finding was in line with those of previous studies, which confirm that *E. coli* is one of the major bacteria that cause diarrhea in sheep, broiler chickens, and cows*.* Moreover, Fouad et al.^42^ and Algammal et al*.*^43^ reported that the *E. coli* prevalence in diarrheic calves was 37.4% and 28.8%, respectively. According to Khalil et al.^44^, 30.2% of the 16 out of 53 sheep rectal swab samples with diarrheal symptoms had positive *E. coli* isolates. Meanwhile, Hafez^45^ found that the *E. coli* prevalence was high at 69.7% in diarrheal sheep. Oppositely, from diarrheal broiler, *E. coli* isolates were found in 40% and 20% of the governorates of El-Fayoum and Giza, respectively, according to EL-Demerdash et al.^46^. A number of variables, including the raising system, the surroundings, the age of the birds, their immunity, and their stage of production, may be responsible for this variance.

The pathogenicity of the *E.* coli strains—their capacity to cause hemolysis and bind to Congo red—was assessed in the existing study. Strains of *E. coli* exhibited both beta- and -alpha hemolysis. Since hemolysis was shown to induce cell membrane damage, it was employed as a phenotypic marker for the pathogenicity factor of *E. coli.* Additionally, 55.3% of the *E. coli* isolates found in diarrheal sheep, broiler chickens, and cows were beta-hemolytic. These almost match the findings of Abd El-Wahed^47^, who reported that 66.7% of the tested isolates of *E. coli* were hemolytic. Furthermore, 44.7% of the entire *E. coli* strains that were recovered from various diarrheal species displayed CR positivity, albeit to varying degrees of redness. Fouad et al.^42^ discovered that, to varying degrees, 60.6% of the *E. coli* isolates under investigation tested positive for CR. Cong red is a straightforward dye that can be added to agar media. Quinn et al.^48^ reported that dye uptake has been shown to be a virulence marker to differentiate between invasive and noninvasive isolates. 44.7% of the total *E. coli* strain recovered from diarrheal sheep, cows, and broiler chickens in the current investigation demonstrated CR positivity, but to varying degrees of red color (40%, 46.7%, and 46.2%, respectively). These findings were not as promising as those of Fouad et al.^42^, who discovered that 60.6% of the tested *E. coli* isolates had varying degrees of CR positivity. The most popular epidemiological marker for classifying pathogenic *E. coli* is thought to be serotyping. Particularly when it comes to *E. coli* that causes diarrhea, some serotypes are known to be closely linked to pathotypes. In order to better understand *E. coli* epidemiology and control the bacteria that cause diarrhea and non-intestinal illnesses, it is more beneficial to analyze the incidence of different *E. coli* serotypes and their distribution patterns across different geographic locations. Eleven *E. coli* isolates were identified using serological analysis. All strains (100%) could be typed.

The most prevalent serogroup was O26:K60 (3), followed by O44:K74(2), O124:K72(2), O25:K11(2), O118:K-(1) and O27:K-(1). When *E. coli* strains were obtained from sheep, serogroups O26:K60 and O44:K74 were found, whereas isolates from cows had O124:K72 and O25:K11, and isolates from chickens had O118:K- and O78:K-. The *E. coli* serogrouping is shown in Table 4. The *E. coli* strains were identified serologically as O157:H7 (n = 4; two isolated from calves and two from goat kids), O125 (n = 3; two isolated from calves and one from lambs), and O44 (n = 3; two isolated from goat kids and one from lambs), according to Abd EL-Tawab et al.^49^ Meanwhile, Algammal et al.^43^ identified seven serogroups (O26, O45, O91, O111, O119, O125, and O128) by serotyping the *E. coli* strains from calf diarrhea. Furthermore, Wilczy´nski et al.^50^ and El-Mongy et al.^51^ reported that serotype O78 was the most common serotype among *E. coli* isolates from all varieties of chickens.

ERIC-PCR profiles in our study allowed us to classify virulent *E. coli* isolates into ERIC-types. Using a 75% similarity limit, dendogram analysis separated them into two large clusters, A and B. Cluster A was split up into A1 and A2 groups. Ninety percent of isolated *E. coli* strains from the two groups A1 and A2 of diarrheagenic sheep were comparable. Cluster B was split up into groups (B1, B2, and B3). There was an 89% similarity between these three groups. Furthermore, for B1, B2, and B3, the corresponding levels of similarity within each group were 96%, 94%, and 92%. The range of identities for all *E. coli* isolates was 0.17 to 1, with corresponding ranges for sheep, cows, and poultry samples (0.67 to 1), (0.22 to 0.6), and (0.67 to 0.17) as shown in Figs. 2 and 3. Sekhar et al.^18^ revealed that ERIC-PCR was demonstrated to be a quick, sharp, and cost-effective fingerprint approach for successful discrimination of *E. coli* isolates based on their genotype. There may be complex transmission of *E. coli* from broiler chickens to cows and the environment, and vice versa, as evidenced by the high DNA fingerprint relatedness shared by several strains of the bacteria from different animals and broiler chickens. Our findings revealed crucial information on the genetic and epidemiological traits of *E. coli* and emphasized the need for stronger biocontainment measures in order to lower the occurrence and effects of the bacteria in animal and poultry husbandry.

The susceptibility pattern of pathogenic *E. coli* to three different disinfectant compounds (TH^4+^, Virkon®S, and H_2_O_2_) was found to be as follows: after 120 min of exposure time at a concentration of 1:100 ml, all testing bacterial strains of *E. coli* were completely sensitive to testing disinfectants TH^4+^ and Virkon®S, while the effectiveness of H_2_O_2_ on *E. coli* isolates was not greater than 50% at 5% concentration after 120 min of exposure. These results were consistent with those of Fawzia et al.^52^, who discovered that *E. coli* isolates were susceptible (86.7%) to Virkon®S (1%) and TH^4+^ (0.2%) using the disc diffusion method. Gehan et al.^53^ found that the synergy of glutaraldehyde and QAC makes TH^4+^ the most potent disinfectant. Additionally, glutaraldehyde-based disinfectants showed a high degree of sensitivity against both *S. aureus* and* E. coli* ^54^. Conversely, Rutala and Weber^55^ indicated that H_2_O_2_ at a concentration of 7.5% was the most effective disinfectant among the oxidizing agents. After five minutes of exposure, R´ıos-Castillo et al.^20^ discovered that H_2_O_2_ integrated with cationic polymers at the same concentration was very effective. According to Lineback et al.^56^, H_2_O_2_ disinfection outperformed quaternary ammonium compounds (QACs) in its ability to destroy *P. aeruginosa* and* S. aureus* biofilms. In this study, following 120 min of exposure, nano zinc oxide was shown to have bactericidal effects on *E. coli* at the maximum dose (0.04 mg/ml). Thus, these results allowed us to investigate the possibility of employing nano zinc oxide to increase hydrogen peroxide's ability to penetrate bacterial cells. It's interesting to note that, in contrast to other concentrations, hydrogen peroxide loaded on ZnO NPs was shown to have a deadly effect against all *E. coli* isolates (100%) at the same concentration and exposure period (0.04 mg/ml and 120 min), whereas the diameter of the NPs ranged from 75.08 to 100.58 nm. Moreover, ZnO NPs have the potential to be antimicrobial effective (average size = 30 nm), causing bacterial cell death by disrupting the integrity of the cell wall^57^. Furthermore, Siddiqi et al.^58^ found that at 125 μg/ml, micro zinc oxide particles had a high level of efficiency against *S. aureus* and* E. coli.* Additionally, the average size of NPs ranged from 5.48 to 34.6 nm. Abdelghany et al.^59^ observed that ZnO NPs had antibacterial activity against various species of bacteria such as *S. aureus*, *E. coli*, and *K*.* pneumonia* with inhibition zones (23.83 ± 0.29, 28.33 ± 0.58, and 23.83 ± 1.04, respectively). ZnO NPs had a biocidal effect by accumulating nanoparticles in the cytoplasm and/or outer bacterial cell wall, which released Zn^2^^+^ and damaged membrane proteins, killing the microbial cell^60,61^. With regards to the SEM image, it displayed randomly distributed ZnO NPs with aggregated particles. Furthermore, peaks at 602.64 cm^−1^ in the FT-IR spectra of the biosynthesized ZnO NPs are linked to the ZnO stretching vibration mode. ZnO NPs' XRD pattern showed a monoclinic structure. The hexagonal phase crystals of zinc oxide were confirmed by the usual diffraction peaks detected at 2θ = 31.72° (100), 34.47 (002), 36.25 (101), 47.71 (102), 56.59 (110), 62.98 (103), and 67.78 (112). The average particle size of ZnO NPs ranged from 12.4 to 18.9 nm^62^.

## Conclusion

The prevalence rate of pathogenic *E. coli* was significantly higher in poultry feces (43.3%) than that of sheep and cows. All *E. coli* isolates from various diarrheagenic animals were recognized using ERIC-PCR, whereas the identities of sheep, cows, and broiler chickens varied from 0.67 to 1.0, 0.22 to 0.6, and 0.67 to 0.17, respectively. Testing *E. coli* strains were particularly susceptible to the disinfectants TH^4+^ and Virkon®S after 120 min of exposure at a dosage of 1:100 ml. The efficacy of H_2_O_2_ against *E. coli* was not greater than 50% at 5% concentration during any testing contact period. It's interesting to note that the H_2_O_2_/ZnO NPs composite exhibits possible antibacterial action against *E. coli* isolates at 0.04 mg/ml after 120 min of exposure. The promising composite proved its stability, and based on their electrophoretic mobility, it had a negative charge of − 0.12 mV, and the hydrodynamic diameter of the particle size was 2625 nm.

## Data Availability

All data generated or analyzed during this study are included in this published article.

## References

[1] Allam SA (2019). Molecular detection of Inva and Hila virulent genes in salmonella serovars isolated from fresh water fish. Slov. Vet. Res..

[2] Eckburg PB (2005). Diversity of the human intestinal microbial flora. Science.

[3] Jang J (2017). Environmental *Escherichia coli*: Ecology and public health implications-a review. J. Appl. Microbiol..

[4] Fairbrother JM, Nadeau E (2006). *Escherichia coli*: On-farm contamination of animals. Rev. Sci Tech..

[5] Arshad R, Farooq S, Ali SS (2006). Manipulation of different media and methods for cost-effective characterization of *Escherichia coli* strains collected from different habitats. Pak. J. Bot..

[6] Kirk MD (2010). World Health Organization estimates of the global and regional disease burden of 22 food-borne bacterial, protozoal, and viral diseases, 2010: A data synthesis. PLoS Med..

[7] Caprioli A, Maugliani A, Michelacci V, Morabito S (2014). Molecular typing of Verocytotoxin-producing *Escherichia coli* (VTEC) strains isolated from food, feed and animals: State of play and standard operating procedures for pulsed field gel electrophoresis (PFGE) typing, profiles interpretation and curation1. EFSA.

[8] Gharieb RM, Fawzi EM, Attia NE, Bayoumi YH (2015). Calf diarrhea in Sharkia province, Egypt: Diagnosis; prevalence, virulence profiles and zoonotic potential of the causative bacterial agents. Int. J. Agric. Sci. Vet. Med..

[9] Ferrari RG, Panzenhagen PHN, Conte-Junior CA (2017). Phenotypic and genotypic eligible methods for Salmonella Typhimurium source tracking. Front. Microbiol. Front. Med. SA.

[10] Nakamura A (2021). Molecular subtyping for source tracking of *Escherichia coli* using core genome multilocus sequence typing at a food manufacturing plant. PLoS One.

[11] Ramadan H (2020). Antimicrobial resistance, genetic diversity and multilocus sequence typing of *Escherichia coli* from humans, retail chicken and ground beef in Egypt. Pathogens.

[12] Chen Y, Perfect JR, White Stefan J, Cantsilieris Stuart (2017). Efficient, cost-effective, high-throughput, multilocus sequencing typing (MLST) Method, NGMLST, and the Analytical Software Program MLSTEZ. Genotyping.

[13] Ramadan AA (2022). Bacterial typing methods from past to present: A comprehensive overview. Gene Rep..

[14] Souza RA, Pitondo-Silva A, Falcão DP, Falcão JP (2010). Evaluation of four molecular typing methodologies as tools for determining taxonomy relations and for identifying species among Yersinia isolates. J. Microbiol. Methods..

[15] Hulton CS, Higgins CF, Sharp PM (1991). ERIC sequences: A novel family of repetitive elements in the genomes of *Escherichia coli*, Salmonella typhimurium and other enterobacteria. Mol. Microbiol..

[16] Chansiripornchai N, Ramasoota P, Sasipreyajan J, Svenson SB (2001). Differentiation of avian *Escherichia coli* (APEC) isolates by random amplified polymorphic DNA (RAPD) analysis. Vet. Microbiol..

[17] Galane PM, Le Roux M (2001). Molecular epidemiology of *Escherichia coli* isolated from young South African children with diarrhoeal diseases. J. Health Popul. Nutr..

[18] Sekhar MS, Sharif NM, Rao TS, Metta M (2017). Genotyping of virulent *Escherichia coli* obtained from poultry and poultry farm workers using enterobacterial repetitive intergenic consensus-polymerase chain reaction. Vet. World..

[19] Mousavi ZE, Fanning S, Butler F (2013). Effect of surface properties of different food contact materials on the efficiency of quaternary ammonium compounds residue recovery and persistence. Int. J. Food Sci. Tech..

[20] Rios-Castillo AG, Gonzalez-Rivas F, Rodriguez-Jerez JJ (2017). Bactericidal efficacy of hydrogen peroxide-based disinfectants against gram-positive and gram-negative bacteria on stainless steel surfaces. J. Food Sci..

[21] Kelly SA (1998). Oxidative stress in toxicology: Established mammalian and emerging piscine model systems. Environ. Health Perspect..

[22] Brayner R (2006). Toxicological impact studies based on *Escherichia coli* bacteria in ultrafine ZnO nanoparticles colloidal medium. Nano Lett..

[23] Cho YI, Yoon KJ (2014). An overview of calf diarrhea - Infectious etiology, diagnosis, and intervention. J. Vet. Sci..

[24] Lee, M.D. & Nolan, K.L. A laboratory manual for the isolation and identification of avian pathogen In: Zavala, L.D., Swayne, D.E., John, R.C., Mark, W.G Wood, J., Pearson, J.E. and Reed, W.M, editors. Editorial, Board for the American Association of Avian Pathologists. 5th ed., Ch. 3. American Association, *Colibacillosis*. P10–16 (2008).

[25] Vidotto MC (1990). Virulence factors of avian *Escherichia coli*. Avian Dis..

[26] Berkhoff HA, Vinal AC (1986). Congo red medium to distinguish between invasive and non-invasive *Escherichia coli* pathogenic for poultry. Avian Dis..

[27] Morris JA, Sojka WJ, Ready RA (1985). Serological comparison of the *Escherichia coli* prototype strains for the F(Y) and Att 25 adhesions implicated in neonatal diarrhoea in calves. Res. Vet. Sci..

[28] Versalovic J, Koeuth T, Lupski JR (1991). Distribution of repetitive DNA sequences in eubacteria and application to fingerprinting of bacterial genomes. Nucleic Acids Res..

[29] Hunter PR (1990). Reproducibility and indices of discriminatory power of microbial typing methods. J. Clin. Microbiol..

[30] Li Q (2008). Antimicrobial nanomaterials for water disinfection and microbial control: potential applications and implications. Water Res..

[31] Salah N (2011). High energy ball milling technique for ZnO nanoparticles as antibacterial material. Int. J. Nanomed..

[32] Clinical and Laboratory Standards Institute (CLSI) Performance standards for antimicrobial susceptibility testing 29^th^ ed: CLSI supplement M100, Wayne, PA. 2019; https://clsi.org/media/2663/m100ed29_sample.pdf

[33] Jianga L (2021). Virulence-related O islands in enterohemorrhagic *Escherichia coli* O157:H7. Gut Microbes..

[34] Cho S (2006). Prevalence and characterization of *Escherichia coli* O157 isolates from Minnesota dairy farms and county fairs. J. Food Prot..

[35] Ateb CN, Mbewe M (2013). Determination of the genetic similarities of fingerprints from *Escherichia coli* O157:H7 isolated from different sources in the North West Province, South Africa using ISR. BOXAIR REP-PCR analysis. Microbiol Res..

[36] Paletta AC, Castro VS, Conte-Junior CA (2020). Shiga toxin-producing and enteroaggregative *Escherichia coli* in animal, foods, and humans: Pathogenicity mechanisms, detection methods, and epidemiology. Curr Microbiol..

[37] Idalia VMN, Bernardo F (2017). *Escherichia coli* as a model organism and its application in biotechnology. Recent Adv. Physiol. Pathog. Biotechnol. Appl. Tech Open Rij. Croat.

[38] Blount ZD (2015). The natural history of model organisms: The unexhausted potential of *Escherichia coli*. Elife.

[39] Aquino MH (2010). Diversity of Campylobacter jejuni and Campylobacter coli genotypes from human and animal sources from Rio de Janeiro. Brazil. Res Vet Sci..

[40] Macedo NR (2011). ERIC-PCR genotyping of Haemophilus parasuis isolates from Brazilian pigs. Vet J..

[41] Munoz V (2011). Phenotypic and phylogenetic characterization of native peanut Bradyrhizobium isolates obtained from Cordoba, Argentina. Syst. Appl. Microbiol..

[42] Fouad H (2022). Prevalence of pathogenic *Escherichia coli* in diarrhoeic cattle calves and antibiotic resistance genes. KVMJ.

[43] Algammal AM (2020). Virulence-determinants and antibiotic-resistance genes of MDR-*Escherichia coli* isolated from secondary infections following FMD-outbreak in cattle. Sci. Rep..

[44] Khalil SA, Eraky MI (2012). Microbiological Study of *Escherichia coli* in Sheep. Alex. J. Vet. Sci..

[45] Hafez AA (2020). Virulence and antimicrobial resistance genes of *Escherichia coli* isolated from diarrheic sheep in the North-West Coast of Egypt. Syst. Rev. Pharm..

[46] EL-Demerdash GO, Fatma A, Heba R (2021). Diarrheic syndrome in broiler and some wild birds caused by *Escherichia coli*. Assiut Vet. Med. J..

[47] Abd-El-Wahed, M. A. Virulence determinant of enterotoxigenic *Escherichia coli* and its relation to adhesive antigen K99 associated with diarrhea in newly born calves. M.V.Sc. Thesis, Zagazig Univ., Fact. Vet. Med (2005).

[48] Quinn, P.J., Carter, M.E., Markey, B.K. & Carter, G.R. Clinical. Veterinary Microbiology. Mosby year book Europe limited, Linton House. London, pp: 109–126 (1994).

[49] Abd El-TA (2020). Prevalence of multi-drug resistant *Escherichia coli* in diarrheic ruminants. BVMJ.

[50] Wilczy’nski J, Stepie’n-Py’sniak D, Wystalska D, Wernicki A (2022). Molecular and serological characteristics of avian pathogenic *Escherichia coli* isolated from various clinical cases of poultry colibacillosis in Poland. Animals.

[51] El-Mongy MA (2018). Serotyping and virulence genes detection in *Escherichia coli* isolated from broiler chickens. J. Biol. Sci..

[52] Fawzia MAA, Hider MHA, Saa'd MSA (2013). In-vitro evaluation by Disc-diffusion and Pits methods of antimicrobial efficiency of disinfectants used in four broiler chicken hatcheries in Babil city/ Iraq. Acad. res. int..

[53] Gehan MZ (2009). In vitro efficacy comparisons of disinfectants used in the commercial poultry farms. Int. J. Poult. Sci..

[54] Singh M (2012). Comparative efficacy evaluation of disinfectants routinely used in hospital practices: India. Indian J. Crit Care Med..

[55] Rutala, W.A. & Weber, D.J. the Healthcare Infection Control Practices Advisory Committee (HICPAC). Guideline for Disinfection and Sterilization in Healthcare Facilities (2008). https://www.cdc.gov/infectioncontrol/guidelines/disinfection/

[56] Lineback CB (2018). Hydrogen peroxide and sodium hypochlorite disinfectants are more effective against Staphylococcus aureus and Pseudomonas aeruginosa biofilms than quaternary ammonium compounds. Antimicrob. Resist. Infect. Control.

[57] Jiang Y, Zhang L, Wen D, Ding Y (2016). Role of physical and chemical interactions in the antibacterial behavior of ZnO nanoparticles against *Escherichia coli*. Mater. Sci Eng: C..

[58] Siddiqi KS, Rahman AU, Tajuddin, Husen A (2018). Properties of Zinc Oxide Nanoparticles and their activity against Microbes. Nanoscale Res. let..

[59] Abdelghany TM (2023). Phytofabrication of zinc oxide nanoparticles with advanced characterization and its antioxidant, anticancer, and antimicrobial activity against pathogenic microorganisms. Biomass Conv. Bioref..

[60] Shi LE (2014). Synthesis, antibacterial activity, antibacterial mechanism and food applications of ZnO nanoparticles: A review. Food Addit. Contam Part A Chem. Anal. Control Expo. Risk Assess..

[61] Dutta RK, Nenavathu BP, Gangishetty MK, Reddy AV (2013). Antibacterial effect of chronic exposure of low concentration ZnO nanoparticles on *Escherichia coli*. J. Environ. Sci. Health A Tox Hazard Subst. Environ. Eng..

[62] Mohamed AA (2021). Eco-friendly mycogenic synthesis of ZnO and CuO nanoparticles for in vitro antibacterial, antibiofilm, and antifungal applications. Biol. Trace Elem. Res..

